# The Role of Organizational Culture in Operating Room Turnaround Time

**DOI:** 10.7759/cureus.1257

**Published:** 2017-05-17

**Authors:** David Ninan, Janet Zhu, Amanda Kore, Elizabeth Wasson, Tricia Fullerton, Barbara Ninan

**Affiliations:** 1 Anesthesiology, Riverside University Health System Medical Center, Moreno Valley, California, United States; 2 School of Nursing, Loma Linda University

**Keywords:** or turn over time, or efficiency, organizational culture

## Abstract

This analysis looks at the application of a robust process improvement methodology to achieve a sustained organizational change. The implementation took place in a safety net hospital’s operating suites that had a problem with relatively long, nonproductive turnover times between surgical procedures. Organizational leadership empowered stakeholders to use Lean and Six-Sigma tools to develop more efficient organizational processes. These processes were then implemented in a phased approach with careful attention to the organization’s culture. The result was a significant reduction in turnover times leading to greater operational efficiency.

## Introduction

The US health care system consumes a disproportionate amount of the nation’s resources. In fact, by the year 2020, the US health care spending is projected to consume 20% of the gross domestic product [1]. In addition to high costs, the health care industry has been plagued with the persistent problem of preventable harm reaching patients. In a push to transform health care safety, The Joint Commission has developed a set of resources called High-Reliability Organizational Assessment and Resources tools available on their website. The change in methodology they propose is called robust process improvement (RPI) to help organizations move toward more efficient and safer health care. RPI is a combination of Lean, Six-Sigma, and change management. Lean and Six-Sigma both address organizational system processes (by decreasing waste and reducing errors), while change management focuses on the organization’s ability to accept, implement, and maintain a change [1].

Change can be challenging for an organization, particularly one that has been operating in a stable environment for a significant period. In cases where change has been achieved, sustaining a new way of doing business can be challenging. Often, new initiatives are met with minimal staff acceptance (i.e., “buy-in”), little effort, and even passive-aggressive sabotage. This report looks at how one organization struggled to improve efficiency and ultimately was successful by implementing a culturally focused change process. While this exercise took place in an operating suite, the techniques and strategies employed are universal and can be applied in many other settings.

## Technical report

The project took place at Riverside County Regional Medical Center (RCRMC), a safety net hospital with 362 beds and 10 operating room (OR) suites. The OR’s average turnover time (TOT) was approximately 45 minutes for elective scheduled surgeries. TOT in an OR represents necessary but non-income generating time in an environment in which the sophisticated equipment and large numbers of highly trained staff lead to high fixed costs. In this case study, the accumulated nonproductive TOT led to significant overtime expenses as well as an increased bottleneck to patient throughput.

The accumulated amount of nonproductive TOT produced significant overtime costs to the institution and a great deal of staff frustration due to the frequent need for after hour work coverage. Multiple attempts to change the practice yielded valuable insight into how to improve the processes, but the changes were not effective in achieving a sustainable change. These failures reinforced the staff’s notion that change was impossible, creating a vicious cycle resulting in decreased staff morale. Many factors were blamed such as a unionized nursing staff or a lack of an adequate computerized infrastructure capable of monitoring these measures. For several reasons, the culture in the OR had devolved to one of apathy about the process and tension between team members.

The OR leadership team decided to target both the inefficiencies and the organizational culture. The first task the leadership team (composed of anesthesia staff and perioperative nurses) undertook was a review of the relative literature [2-3] and a collaborative analysis of what the causal factors were in the prior failed initiatives. The team concluded the failure point was not in diagnosing ways to improve efficiency but, rather, how these changes were implemented. This guided the decision that the priority should be in achieving maximal front-line staff buy-in.

Building on the above premise, a group of stakeholders who were deemed to be early adopters were assembled to work on the problem. The OR leadership facilitated the discussions using established Lean and Six-Sigma tools while encouraging the group’s autonomy in finding ways to make each provider’s job simpler and more efficient. The process began by mapping out the current flow of operations between surgeries. They then looked for areas of redundancy and eliminated aspects such as multiple teams asking the patient the same questions (unless it was safety related). They then looked for easy areas to change like implementing pre-filled syringes to eliminating steps in the overall processes.

The area that made the biggest impact was where a serial flow pattern could be changed to one of parallel processing. For example, in the prior workflow, after one surgical procedure, the surgeon, anesthesiologist, and nurse would all converge on the next patient to prepare that patient for the coming surgical procedure. The flow was changed from the majority of the work being done between operations to a more parallel process. In the new flow, a member of the nursing, surgical, and anesthesia teams would see the patient while the prior surgical procedure was ongoing. That way, at the conclusion of the prior operation, they would only need to verify important elements and, thus, significantly reduce the time needed for processing the patient. In addition to seeing the patient, they would identify any special needs, equipment, or other materials that may not have been uncovered in the scheduling process. The net result was that, during the prior case, the patient was largely processed and ready to go.

After streamlining the processes, the team was directed to identify patient movement at each stage in the process. Theoretically, the major factors that pushed or pulled a patient through the system would be a primary driver in reducing the TOT. Based on the best practice workflow, 20 minutes would be sufficient if the process worked smoothly. Thus, 20 minutes after the prior patient left a specific OR suite, the next patient would be wheeled back. If there was a delay in the system (e.g., an item was not sterilized correctly), the individual responsible for the delay would communicate this to the party who transported the patient into the OR suite. To ensure accurate data collection, all delay reasons were verbalized by the OR suite’s staff before the start of the surgical procedure, and the reason was entered into the tracking system for future investigation and analysis.

The team then developed a method to measure the process continually. TOT was defined as the time it took the OR staff to go from one scheduled operation to another (i.e., “wheels out” to “wheels in”). The team wanted a clearly defined measurement specific to their performance and thus chose to exclude all factors outside the control of anesthesia or nursing. For this reason, prolonged TOT’s were excluded if they were due to factors such as the patient or surgeon being late on arrival or emergent operations where the staff did not have time to prepare in advance. The goal of the reporting structure was to develop an accurate measure of staff performance over which they had direct control.

The final phase was to implement the changes while maximizing staff buy-in. A phased implementation approach would both uncover and solve problems on a smaller scale and help the entire team acclimate to the changes. The process was implemented for a full month in one OR suite per day as a pilot study. The pilot staff was carefully selected to be composed of individuals deemed both highly respected by their peers and have a history of being early adopters to prior changes. After each workday, the process was discussed in a debriefing session and opportunities for improvement were sought. In addition, the individual team members were praised for their ideas and efficient work. Gradually, the process was more widely implemented to include slower adopters who often had reservations about the process. Concerns were addressed on a one-on-one basis with an emphasis on valuing the person’s input and reassuring the concerned individual of the goal to make everyone’s job run smoother and more efficient. Close monitoring and frequent coaching were utilized where appropriate. It is possible that the approach of the leadership to provide individualized attention to the staff helped them feel like valued team members, thus helping them be more open to change.

## Discussion

An ideal TOT is difficult to determine partly due to the great number of variables. Foster published a paper that combined data from various ORs in the United States in an attempt to develop benchmark metrics [4]. The benchmark TOTs are depicted as follows: median, 28.5 minutes; 90th percentile, 22.7 minutes; and 95th percentile, 21.4 minutes.

When the process was officially implemented in full at RCRMC, nearly all staff had participated in the pilot project and had the opportunity to give individual feedback. The first quarter results of the initiative yielded an average TOT of 21.7 minutes, which was publically posted and the staff was praised for their performance.

The team was provided weekly bulletin board performance reports relative to external benchmarks. Throughout the process, there were no disgruntled workers who approached management with frustrations, which may be due to the group feeling a sense of pride in their accomplishments. The process outcomes were sustained and achieved an average TOT of 22.7 minutes.

## Conclusions

This report illustrates the application of a robust process improvement initiative that emphasized the importance of addressing the culture in a change implementation procedure. It highlights the importance of investing in human factors such as the initial buy-in of key team members, the value of a phased implementation, and the importance of frequent feedback with positive reinforcement. This approach to implementing change has broad application to many settings within the health care system.
